# Increased prevalence of vulnerable atherosclerotic plaques and low levels of natural IgM antibodies against phosphorylcholine in patients with systemic lupus erythematosus

**DOI:** 10.1186/ar3193

**Published:** 2010-11-23

**Authors:** Cristina Anania, Thomas Gustafsson, Xiang Hua, Jun Su, Max Vikström, Ulf de Faire, Mikael Heimbürger, Tomas Jogestrand, Johan Frostegård

**Affiliations:** 1 Department of Medicine, Karolinska University Hospital, Huddinge, 141 86 Stockholm, Sweden; 2 Karolinska Institutet, 171 77 Stockholm, Sweden; 3 Department of Clinical Physiology, Karolinska University Hospital, Huddinge, 141 86 Stockholm, Sweden; 4 Division of Cardiovascular Epidemiology, Institute of Environmental Medicine, 171 76 Stockholm, Sweden; 5 Department of Cardiology, Karolinska University Hospital, Solna, 171 76 Stockholm, Sweden

## Abstract

**Introduction:**

The risk of cardiovascular disease (CVD) and atherosclerosis is reported to be increased in systemic lupus erythematosus (SLE). We recently reported a negative association between natural IgM-antibodies against phosphorylcholine (anti-PC) in the general population, high anti-PC levels leading to decreased atherosclerosis development and low levels to increased risk of CVD. Potential mechanisms include anti-inflammatory properties and inhibition of uptake of oxidized low density lipoprotein (LDL) in macrophages. The objective herein was to study atherosclerosis in SLE in detail and in relation to traditional and non-traditional risk factors.

**Methods:**

A total of 114 patients with SLE were compared with 122 age- and sex-matched population-based controls. Common carotid intima-media thickness (IMT), calculated intima-media area (cIMa) and plaque occurrence were determined by B-mode ultrasound as a surrogate measure of atherosclerosis. Plaques were graded according to echogenicity and grouped as 1 to 4, with 1 being echoluscent, and considered most vulnerable. Anti-PC was studied by ELISA.

**Results:**

Hypertension, triglycerides and insulin resistance (determined by homeostasis model assessment of insulin resistance) and C-reactive protein (CRP) were increased in SLE (*P *< 0.01) while smoking, LDL, high density lipoprotein (HDL) did not differ between groups. Low levels of anti-PC IgM (lowest tertile) were more common in SLE patients than in controls (*P *= 0.0022). IMT and cIMa did not differ significantly between groups. However, plaques were more often found in SLE patients (*P *= 0.029). Age, LDL and IgM anti-PC (lowest tertile) were independently associated with plaque occurrence in SLE. Further, in the left carotid arteries echoluscent plaques (grade 1) were more prevalent in SLE as compared to controls (*P *< 0.016).

**Conclusions:**

Plaque occurrence in the carotid arteries is increased in SLE and is independently associated with age, LDL and low anti-PC levels. Vulnerable plaques were more common in SLE. Anti-PC could be a novel risk marker also with a therapeutic potential in SLE.

## Introduction

Early studies suggested that there is a bimodal pattern in SLE, with manifestations including nephritis occurring early and cardiovascular disease (CVD) later in life [1]. Several case-control studies indicate that atherosclerosis is increased in SLE [2-5]. It has ever since become clear that the risk of CVD is increased in SLE [6], which is a clinical problem and also theoretically interesting since atherosclerosis, the major cause of CVD, largely can be considered an inflammatory disease where the immune system may play an important role [7]. Activated macrophages and T cells producing inflammatory cytokines are present in the atherosclerotic lesions [8]. Oxidized low density lipoprotein (oxLDL) may play a major role in atherosclerosis, constituting much of the lipid moiety present in lesions. In addition, oxLDL has immune stimulatory and pro-inflammatory properties [9,10]. The pro-inflammatory effects of oxLDL may be caused by inflammatory phospholipids with platelet activating factor (PAF)-like properties where phosphorylcholine (PC) plays a major role in binding to the PAF-receptor [11,12]. We recently demonstrated that natural IgM antibodies against PC (anti-PC) are negatively associated with atherosclerosis development in humans [13] and that low levels of anti-PC predict increased CVD risk [14-17]. Further, we reported that anti-PC were decreased in a nested case-control SLE study and that anti-PC has anti-inflammatory effects relevant in both atherosclerosis and SLE, inhibiting the effects of an inflammatory phospholipid, PAF [17], which is increased in active SLE [18].

Thus, a combination of traditional and non-traditional risk factors may account for the high prevalence of CVD in SLE including dyslipemia, hypertension, oxLDL, anti-phospholipid antibodies (aPL) and raised activity of inflammatory factors like TNF and PAF-acetylhydrolase (LDL-PLA2), C-reactive protein (CRP) [5,19-22].

We here report that atherosclerotic plaques are more common and of potentially lower stability in SLE patients as compared to controls and that among other factors, atheroprotective anti-PC are implicated. The implications of these findings are discussed.

## Materials and methods

### Study group

The study group consisted of 114 patients from Karolinska University Hospital Huddinge with diagnosed SLE and 122 sex- and age-matched population-based controls. Altogether, 160 patients younger than 70 years with SLE were identified in the year 2006 through a careful survey of patient journals of all patients admitted to Huddinge Hospital for suspect SLE or SLE. Of these, 122 initially, but finally only 118, agreed to participate and were included in our study which was named SLEVIC (SLE Vascular Impact Cohort) study. One hundred twenty-two age- and sex-matched controls (recruited randomly from Huddinge catchment area) were accepted to participate. The inclusion was initiated in August 2006 and ended in December 2007. Four patients more where excluded because they did not fulfil the American College of Rheumatology (ACR) criteria. Of these 114 patients, three missed the ultrasound investigation of carotids. Finally, our study consisted of data for 114 patients fulfilling the 1982 revised criteria of the ACR for SLE and 122 sex- and age-matched controls. The study was approved by the Karolinska Institute research ethics committee and is in accordance with the Helsinki Declaration. All subjects gave informed consent before entering the study.

### Study protocol

The investigation included a written questionnaire, an interview, and a physical examination by a rheumatologist, laboratory determinations, and ultrasound examination of the carotid arteries. SLE activity was determined with the Systemic Lupus Activity Measure (SLAM) and also with Systemic Lupus Erythematosus diseases activity index (SLEDAI). Organ damage was determined with Systemic Lupus International Collaborating Clinics (SLICC) damage index.

### Assays

Blood samples were collected between 07.30 and 10.00 h after an overnight fast. The biochemical variables were determined by standard laboratory methods. Serum and cells were separately prepared in the laboratory before storage at -80°C.

Immunological analyses of anticardiolipin antibodies (aCL) and beta-2-glycoprotein antibodies were run by the Karolinska Immune lab, Solna by an enzyme-linked immunosorbent assay (ELISA). Lupus anticoagulans was determined using nefelometri.

Anti-PC IgM was determined by use of a commercial kit (Athera CVDefine-TM, Stockholm, Sweden) as described by the manufacturers. Anti-PC IgG was determined by use of the CVDefine kit, but the secondary antibody was switched to detect IgG (HRP-goat anti-Human IgG, Invitrogen, Sweden).

Insulin resistance was assessed by calculating the homeostasis model assessment of insulin resistance (HOMA_IR) using the formula fasting insulin (μU/ml)*fasting glucose (mmol/L)/22.5

### Carotid B-mode ultrasonography

The right and left carotid arteries were examined with a duplex scanner (Sequoia, Siemens Acuson, Mountain View, Ca, USA) using a 6 MHz linear array transducer. The patients were investigated in the supine position with the head slightly turned from the sonographer. Two trained sonographers performed all scans. The carotid arteries were carefully examined with regard to wall changes. The far wall of the common carotid artery (CCA), 0.5 to 1.0 cm proximal to the beginning of the carotid bulb, was used for measurements of the intima-media thickness (IMT). The IMT was defined as the distance between the leading edge of the lumen-intima echo and the leading edge of the media-adventitia echo. The CCA lumen diameter was defined as the distance between the leading edge of the intima-lumen echo of the near wall and the leading edge of the lumen-intima echo of the far wall. The examinations were digitally stored for subsequent analyses by a computer system [23] with automated tracing of echo interfaces and measurements of distances between the wall echoes within a 10 mm long section of CCA in late diastole, defined by a simultaneous electrocardiographic recording. The mean values of the IMT and lumen diameter within the 10 mm long section were calculated. When a plaque was observed in the region of the CCA measurements, the IMT was not measured.

Carotid plaque was defined as a localized intima-media thickening of greater than 1 mm and at least a 100% increase in thickness compared with adjacent wall segments. Plaque was screened for in the common, internal and external carotid arteries. Plaque occurrence was scored as the absence of plaque, the presence of unilateral plaque, and the presence of bilateral plaque. Plaque morphology in terms of echogenicity was assessed in a modified version of the classification proposed by Gray-Weale *et al. *[24] and graded from 1 to 4 as echolucent, predominantly echolucent, predominantly echogenic and echogenic. Echolucency was defined with the arterial lumen as reference and echogenicity with the far wall adventitia as reference.

The differences between repeated measurements of IMT and lumen diameter, by using the automated analyzing system, were 4.9% and 2.4% (coefficient of variation), respectively (with an IMT of 0.44 to 1.02 mm and a lumen diameter of 4.38 to 7.9 mm). To compensate for the stretching effect of arterial distension (secondary to increased arterial pressure) on the wall thickness, the cross-sectional intima-media area was calculated by using the formula 3.14 ((lumen diameter/2 + intima-media thickness)^2 ^- (lumen diameter/2)^2^). This calculated intima-media area (cIMa), but not the IMT, has been shown to be unaffected by variations in artery distension secondary to changes in blood pressure [25]. The ultrasonographic methods used have been described in detail previously [26,27]. Repeated classification of plaque morphology showed a correlation coefficient of 0.7 (*P *< 0.05) between the first and second classification (*n *= 50).

### Anthropometrical assessments

Body mass index (BMI) was calculated from weight/height^2 ^(kg/m^2^). Waist circumference (WC) was measured to the nearest 0.5 cm midway between the iliac crest and the lower rib margin. Hip circumference (HC) was measured in the horizontal plane around the symphysis pubis.

### Statistical methods

Determinations were dichotomized or determined as continuous variables as indicated. We calculated percentiles based on distributions in the whole study group. Age, gender and geography were matched for by the design of the study. Data are presented as means (with 95% confidence intervals, CI) or medians (with interquartile ranges, IQR) depending on their distribution. Comparisons between groups were made with the Mann-Whitney U-test, median test or Student's *t*-test. In order to establish the association between potential risk factors for atherosclerosis and atherosclerotic plaque logistic regression was applied with adjustment for covariates. SAS was used for the statistical analyses (release 9.1, SAS Institute Inc., Cary, NC, USA).

## Results

The characteristics of patients and controls are presented in Table 1. Major differences between cases and controls include increased prevalence of: hypertension (*P *< 0.001), defined as blood pressure ≥ 140/90 and/or treatment against hypertension. Further, in SLE we determined decreased levels of LDL (*P *= 0.026) and increased levels of TG (*P *= 0.003), CRP (*P *< 0.001) and HOMA-IR (*P *= 0.011). Low anti-PC IgM- levels occurred more often in patients (lowest tertile; *P *= 0.002; anti-PC determinations available in 111 SLE-cases and 118 controls). There were no significant differences in smoking, diabetes (few cases) or BMI.

**Table 1:** Characteristics of the study groups

	Cases	Controls	*P*-level
Number	114	122	
Age. year	47.94 ± 13.17	49.11 ± 12.68	0.45
Male gender, % (no.)	12.28% (*n *= 14)	10.65% (*n *= 13)	0.94
Current smokers,% (no.)	14.03% (*n *= 16)	15.57% (*n *= 19)	0.47
Presence of diabetes,% (no)	5.26% (*n *= 6)	2.45% (*n *= 3)	0.22
Presence of hypertension, % (no.)	57.89% (*n *= 66)	26.22% (*n *= 32)	<0.001
LDL>3 mmol/L,% (no.)	27.19% (*n *= 31)	44.26% (*n *= 54)	0.006
Current statins,% (no.)	10.52% (*n *= 12)	4.09% (*n *= 5)	0.048
Total cholesterol, mmol/L	4.7 ± 1.1	4.8 ± 1.0	0.23
LDL, mmol/L	2.5 ± 0.88	2.8 ± 0.80	0.026
HDL, mmol/L	1.6 (1.3 to 1.8)	1.6 (1.3 to 1.9)	0.31
Triglycerides, mmol/L	0.99 (0.7 to 1.4)	0.78 (0.55 to 1.10)	0.003
CRP, mg/L	4.44 (0.8 to 4.8)	2.04(0.5 to 2.5)	<0.001
Apolipoprotein E	42.69 ± 14.7	38.84 ± 12.05	0.033
ApoB/ApoA1	0.55 (0.50 to 0.70)	0.60 (0.50 to 0.70)	0.39
BMI (kg/m2)	24.89 (20.96 to 27.85)	24.67 (22.41 to 27.82)	0.58
HOMA IR	1.34 (0.80 to 1.96)	1.05 (0.69 to 1.48)	0.011
IgM antiPC. U/ml	61.28 (36.44 to 176.06)	93.74 (52.90 to 134.22)	0.025
IgG antiPC. U/ml	9.36 (5.45 to 14.76)	7.45 (4.62 to 11.66)	0.0083
IgM antiPC < = 10^th ^percentile (%)	16.5%	2.8%	< = 0.0005
IgM antiPC < = 25^th ^percentile (%)	32.1%	17.9	0.016
IgM antiPC < = 33th percentile (%)	42.0%	22.6%	0.0022

anti-PC, antibodies against PC; CR, C-reactive protein; HDL, high density lipoprotein; HOMA, homeostasis model assessment of insulin resistance; LDL, low density lipoprotein; PC, phosphorylcholine.

In Table 2, SLE-patients and controls are compared for atherosclerosis-related measurements. While IMT and cIMa did not differ between groups, we determined a difference in occurrence of atherosclerotic plaques (*P *= 0.029). Further, left-sided echoluscent plaques were more prevalent in SLE as compared to controls (*P *< 0.016) but there was no significance at the right side. CVD occurrence was increased in SLE (*P *< 0.01) when CVD was defined herein as a history of cerebrovascular events, acute coronary syndrome (ACS), coronary artery by-pass graft (CABG), heart valve prothesis/impairment, peripheral arterial surgery or claudication.

**Table 2:** Cardiovascular measurements in SLE-patients and controls

	Cases	Controls	*P*-level
IMT R, mm	0.60 ± 0.13	0.62 ± 0.13	0.27
IMT L, mm	0.59 (0.50 to 0.71)	0.60 (0.52 to 0.70)	0.62
Plaque,% (no.)	42.98% (*n *= 49)	30.32% (*n *= 37)	0.029
cIMarea R, mm^2^	11.39 (9.60 to 14.34)	11.90 (9.9.93 to 14.01)	0.51
cIMarea L, mm^2^	11.81 (9.41 to 13.43)	11.64 (9.88 to 13.83)	0.78
**Low-echogenic plaques (grade 1) left and right carotid artery**	**44**	**31**	**0.0962**
Low-echogenic plaques (grade 1) left carotid artery	25	13	0.016
Low-echogenic plaques (grade 1) right carotid artery	19	18	0.62
Plaque distribution*	Plaque 0 = 62 (54.38%)	Plaque 0 = 85 (69.67%)	0.015
	Plaque 1 = 20 (17.54%)	Plaque 1 = 20 (16.39%)	0.74
	Plaque 2 = 29 (25.43%)	Plaque 2 = 17 (13.93%)	0.019
History of cerebrovascular events	7.89% (*n *= 9)	0.81% (*n *= 1)	0.007
History of AMI	4.38% (*n *= 5)	0	0.025
History of CABG	2.63% (*n *= 3)	0	-
History of heart valve prothesis/impairmeant	9.64% (*n *= 11)	0.81% (*n *= 1)	0.002
History of peripheral arterial surgery	1.75% (*n *= 2)	0	-
Claudication	8.77% (*n *= 10)	0.81% (*n *= 1)	0.003
CVD**	21.92% (*n *= 25)	2.45% (*n *= 3)	< 0.001

*Plaque 0, no plaque; Plaque 1, plaque on one side; Plaque 2, plaque on both sides.

**Either of history of: cerebrovascular events, AMI, CABG, heart valve prothesis/impairment, peripheral arterial surgery, claudication.

AMI, acute myocardial infarction; CABG, coronary artery by-pass graft; cIMa, calculated intima-media area; CVD cardiovascular disease; IMT, common carotid intima-media thickness.

In Table 3, SLE-patients with or without plaques are compared. In univariate analysis, age (*P *< 0.001), SLE duration (*P *= 0.025), hypertension (*P *= 0.014), fasting glucose-level (*P *= 0.001) but not HOMA insulin resistance, LDL-cholesterol (*P *< 0.001), total cholesterol (*P *< 0.001), apoB/apoA1 (*P *= 0.004), BMI (*P *= 0.032), and anti PC-levels (as determined both as low vs high levels and as continuous values) were significantly different between SLE-patients with and without plaques. The total intake of glucocorticoid medications was not associated with atherosclerotic plaques.

**Table 3:** Comparison between SLE patients with and without atherosclerotic plaques

	SLE patients		
	
	With plaque (*n *= 49)	Without plaque (*n *= 62)	*P*
Age, years	55.79 ± 9.23	41.7 ± 12.76	< 0.001
Disease duration, mean	14.00 years	9.82 years	0.025
Current smokers, % (no.)	7.2% (*n *= 8)	6.3% (*n *= 7)	0.44
Presence of hypertension,% (no.)	31.5% (*n *= 35)	27% (*n *= 30)	0.014
Presence of diabetes,% (no.)	4.5% (*n *= 5)	0.9% (*n *= 1)	0.047
HOMA IR	1.41 (0.91 to 1.94)	1.20 (0.73 to 1.94)	0.41
Glucos (mmol/L)	4.70 (4.30 to 5.00)	4.35 (3.90 to 4.70)	0.0013
LDL(mmol/L)	2.87 ± 1.05	2.28 ± 0.66	0.001
Hyperlipidemia (LDL > 3)	38.77 (*n *= 19)	19.35 (*n *= 12)	0.023
Kolesterol (mmol/L)	5.05 ± 1.26	4.35 ± 0.98	0.001
ApoB/ApoA1, median	0.60 (0.50 to 0.80)	0.50 (0.45 to 0.60)	0.004
High sensitivity CRP, mg/l	1.85 (0.82 to 4.05)	1.75 (0.69 to 4.8)	0.980
Triglicerides (mmol/L)	1.00 (0.74 to 1.50)	0.94 (0.61 to 1.30)	0.065
BMI (kg/m2)	25.95 (22.28 to 28.41)	24.16 (20.08 to 26.84)	0.032
SLAM	6.00 (5 to 9)	6.50 (4 to 10)	0.41
SLEDAI	2 (0 to 5)	2 (0 to 6)	0.46
SLICC	1 (0 to 4)	1(0 to 2)	0.39
Lupus anticoagulans	10.81% (*n *= 12)	14.41% (*n *= 16)	0.87
Anticardiolipin Ab	8.18% (*n *= 9)	11.81% (*n *= 13)	0.70
Beta 2 glycoprotein 1	15.45% (*n *= 17)	18.18% (*n *= 20)	0.83
Cumulative lifetime glukocorticoid dose/year (g/year)	2.49 (1.75 to 3.68)	2.59 (1.87 to 3.49)	0.952
Glucocorticoid last year, gram	0.9 (0 to 2.19)	1.8 (0.07 to 2.70)	0.093
Chloroquin/hydroxychlorochine	42.85% (*n *= 21)	54.83% (*n *= 34)	*P *= 0.210
IgM antiPC	48.75 (25.79 to 104.78)	85.94 (50.02 to 231.52)	*P *= 0.001
IgM antiPC < = 10^th ^percentile (%)	23.40	10.17	0.0651
IgM antiPC < = 25^th ^percentile (%)	40.43	23.73	0.0651
IgM antiPC < = 33^th ^percentile (%)	57.45	28.81	0.0030

anti-PC, antibodies against PC; CR, C-reactive protein; HDL, high density lipoprotein; HOMA, homeostasis model assessment of insulin resistance; LDL, low density lipoprotein; PC, phosphorylcholine; SLAM, Systemic Lupus Activity Measure; SLEDAI, Systemic Lupus Erythematosus diseases activity index; SLICC, Systemic Lupus International Collaborating Clinics damage index.

In a multivariate analysis we included only factors in the model which were independently associated with plaque prevalence in preceding univariate analyses. Furthermore, if the same type of interrelated factors were univariately associated, only the strongest was included. As indicated in Table 4, only age, hyperlipidemia (LDL > 3 mmol/L) and anti-PC IgM (below lowest tertile) remained significant and independently associated with plaque occurrence.

**Table 4:** Multiple regression analysis in relation to occurrence of atherosclerotic plaques in SLE

Explanatory variable	OR	95% CI	*P*-value
Age	7.443	2.45122.602	0.0004
Hypertension	1.484	0.527 4.176	0.4550
Hyperlipidemia (LDL > 3 mmol/l)	5.012	1.511 16.624	0.0084
Glucose	2.299	0.886 5.964	0.0869
IgM anti-PC < = 51.19	2.920	1.080 7.895	0.0348

anti-PC, antibodies against phosphorylcholine; LDL, low density lipoprotein.

If patients with previous CVD are excluded, the associations between low anti-PC and plaque prevalence remain significant (OR 4.4, CI 1.34 to 14.88, *P*= 0.029).

## Discussion

We here report that patients with SLE from a novel cohort in Huddinge, Southern Stockholm had an increased number of carotid atherosclerotic plaques as compared to sex- and age-matched controls from the same area. However, common carotid IMT and cIMa did not differ between groups. The observation of increased occurrence of atherosclerotic plaques is in accord with other, recent observational and/or prospective studies [2,20] and controlled studies [4,28], where increased atherosclerosis has been reported as a feature in SLE.

The somewhat surprising finding that plaque occurrence but not IMT or cIMa is increased in SLE is in line with a previous publication where IMT was even decreased in SLE [4]. Furthermore, in the present study patients with SLE had echolucent plaques more often than controls in the left carotid artery. Carotid echolucent plaques are considered to represent more vulnerable atherosclerotic lesions than echogenic plaques [29]. Thus, individuals with carotid stenoses and echolucent plaques were reported to have an increased risk of stroke and cerebrovascular events compared with individuals with stenoses and more echogenic plaques [30]. The reason for the increased occurrence of echolucent plaques on the left but not on the right side in SLE-patients is not clear. However, a difference between the left and right side concerning carotid atherosclerosis has been reported previously. Thus, in one study of borderline hypertension we found a significantly thicker intima-media on the left than on the right side [26]. This observation has been confirmed by others [31,32]. Our present and previous findings raise the possibility that atherosclerotic lesions develop in different pace and mode in the left and right carotid arteries, possibly because of the difference in gross anatomy of the left and right CCA which might create different shear stress conditions. Shear stress has been shown to be related to both intima-media thickness and echogenecity [33]. Another explanation for the observed side differences is, of course, that they may be a result of chance.

We here report that low levels of natural IgM anti-PC antibodies are more prevalent in a cohort of SLE-patients as compared with controls. We recently reported similar findings in a nested case-control study, where SLE patients with CVD were compared to those without CVD and with controls [34], and in this study, low anti-PC IgM was reported in line with our present observation [34]. We also noted that anti-PC IgG was higher in SLE-patients than in controls in the present study, which did not accord with our previous data [34]. However, the studies differ in important ways. Our previous study was designed to determine risk factors of CVD and not atherosclerosis, and was designed as a nested-case control study, where SLE-cases with CVD were the basis of selection. Thus, the age was much higher in this study, and there were no male SLE-patients. It should be emphasized that prospective studies (and further experimental studies) are needed to establish a mechanistic role for anti-PC IgG (and IgM), and it is possible that the role of especially anti-PC IgG may differ in different stages of disease. Of note, anti-PC IgM appears to be more important for atherosclerosis than IgG [13]. It is furthermore possible that there may be important differences between the adaptive immune response, which is commonly of IgG isotype, and the natural immune response, as represented by anti-PC IgM. Whether dysregulation of natural immunity in SLE where anti-PC IgM (which are anti-inflammatory [34]) could play a role in the pathogenesis of SLE is an interesting possibility which deserves further study.

Anti-PC IgM was negatively but non-significantly associated with SLICC, and larger studies are needed to establish whether such an association is significant and could play a role in SLE.

We recently reported in different population-based cohorts that low IgM anti-PC is an independent and novel risk marker for development of cerebro- and cardiovascular disease in the general population [14-16] and also that it could predict death in uremic patients treated with hemodialysis [35]. Thus, low anti-PC IgM could represent an immune deficient state, predisposing to CVD and in principle also to SLE, since anti-PC has anti-inflammatory properties, inhibiting inflammatory phospholipids as platelet activating factor [34]. Interestingly, the strongly pro-inflammatory lipid mediator PAF is implicated in active SLE [18] and a decreased capacity to inhibit such effects could predispose to symtoms of SLE or SLE itself.

Further, we demonstrated that human anti-PC inhibit uptake of oxLDL in macrophages, implying another non-mutually exclusive possible mechanism by which anti-PC IgM could be atheroprotective [15].

While information about the role of natural antibodies as anti-PC in humans and in clinical conditions has been scarce, more is known in mice-models, where anti-PC IgM protect against lethal meningococcal infections [36].

We reported that IgM anti-PC levels are higher in a population from New Guinea than among Swedish age- and sex-matched controls. These individuals live a traditional life as horticulturalists where CVD rheumatic diseases appear to be rare, a finding that is not likely to be caused by a decreased life expectancy in adulthood [37]. We hypothesized that this may contribute to their low incidence of CVD, in addition to more favourable metabolic and other risk factors, except smoking, surprisingly enough. Diet factors and exposure to infectious agents including nematodes and parasites may contribute, but the reasons for differences in anti-PC levels among cohorts are not well understood [38]. The possibility that diet factors contribute to anti-PC levels is supported by our recent findings in patients with rheumatoid arthritis, where a gluten-free vegan diet included an increase [39], which was also the case in another study where patients on a self-reported Mediterranean diet had raised levels [40]. It is also possible that anti-PC levels are influenced by consumption into tissue, for example because of increased apoptosis and thus binding of anti-PC.

Among traditional risk factors, hypertension and increased triglycerides was significantly more common in SLE, while smoking was not, which in general is in line with previous observations [3,5]. LDL is not known to be commonly raised in SLE, and in this study, the frequency of hyperlipidemia was even higher among controls. An interesting finding is that HOMA, a measure of insulin resistance, was increased in SLE. This confirms findings in a previous report [41] and implies that SLE in general is characterized by early metabolic changes.

Within the SLE group, plaque occurrence was independently associated with age and LDL (positively) and negatively with anti-PC IgM. This finding is in accordance with our previous study where we reported a negative association between IgM anti-PC and atherosclerosis development in hypertensives [13]. Thus, traditional risk factors in combination with low levels of anti-PC IgM may explain the observed increased occurrence of plaques in SLE as observed in the present study.

Cumulative or present doses of prednisolone (or corticosteroids) were not associated with the occurrence of atherosclerotic plaques. Prednisolone has been much discussed in rheumatic disease and has often been described as pro-atherogenic due to its unfavourable effects on metabolic factors. However, we recently reported that in rheumatoid arthritis, there is no association between prednisolone intake and atherosclerosis development [42]. Our present data argue against the possibility that prednisolone is proatherogenic in SLE. It is possible that the anti-inflammatory effects of corticosteroids are more important than the negative influence on lipids in relation to atherosclerosis [43].

## Conclusions

Taken together, our data indicate that atherosclerotic plaques, but not general atherosclerosis as indicated by IMT measurements, are more prevalent in SLE as compared to controls. Further, vulnerable atherosclerotic plaques are more common in SLE. Age, LDL and low levels of anti-PC IgM are independently associated with the prevalence of atherosclerotic plaques in SLE.

## Abbreviations

ACS: acute coronary syndrome; Anti-PC: antibodies against PC; aPL: anti-phospholipid antibodies; BMI: body mass index; cIMa: calculated intima-media area; CABG: coronary artery by-pass graft; CCA: common carotid artery; CI: confidence interval; CRP: C-reactive protein; CVD: cardiovascular disease; HC: hip circumference; HDL: high density lipoprotein; IM: intima-media; IMT: intima-media thickness; IQR: interquartile range; LDL: low density lipoprotein; oxLDL: oxidized low density lipoprotein; PAF: platelet activating factor; PC: phosphorylcholine; SLE: systemic lupus erythematosus; WC: waist circumference.

## Competing interests

JF and UdF are named as co-inventors on patents relating to anti-PC, owned by Athera Biotechnologies where they are minor share holders.

## Authors' contributions

CA played a major role in collecting and managing the database, analysing statistics with JF and MV, and participating in drafting of the manuscript. TG performed and analysed physiological investigations with TJ, and participated in drafting the manuscript. XH and JS performed and analysed experimental data, and participated in drafting the manuscript. MV played a major role in statistical analyses, and participated in drafting the manuscript. UdF participated in statistical analyses, participated in drafting of the manuscript and design of study. MH participated in data collection, drafting the manuscript and the design of study. TJ performed and analysed physiological investigations with TG, and participated in drafting the manuscript. JF played a major role in design of study, drafting the manuscript, statistical analyses and participated in data collection.
